# Sexually Transmitted Infections and Associated Factors in Homosexuals and Bisexuals in Granada (Spain) during the Period 2000–2015

**DOI:** 10.3390/ijerph16162958

**Published:** 2019-08-16

**Authors:** Isabel Llavero-Molino, María Teresa Sánchez-Ocón, María Ángeles Pérez-Morente, Beatriz Espadafor-López, Adelina Martín-Salvador, Encarnación Martínez-García, César Hueso-Montoro

**Affiliations:** 1 Hospital La Axarquía, Andalusian Health System, 29740 Vélez-Málaga, Spain; 2 University Hospital Virgen de las Nieves, Andalusian Health System, 18014 Granada, Spain; 3 Faculty of Health Sciences, University of Jaén, 23071 Jaén, Spain; 4 Sexually Transmitted Infection Clinic, Andalusian Health System, 18012 Granada, Spain; 5 Faculty of Health Sciences, University of Granada, 52005 Melilla, Spain; 6 Faculty of Health Sciences, University of Granada, 18016 Granada, Spain

**Keywords:** sexually transmitted diseases, risk factors, sexual and gender minorities

## Abstract

Sexually transmitted infections (STIs) are a major public health issue. Previous research shows the vulnerability of the homosexual and bisexual population, as well as the influence of economic, political, and cultural determinants. The aim of this study was to describe the socio-demographic healthcare profile and the main risk factors associated with STIs in homosexuals and bisexuals seen at the STI clinic in Granada (Spain) during the years 2000–2015. Infection prevalences were compared between the economic crisis period (2008–2014) and the rest of the years analysed. A cross-sectional observational and analytical study was conducted by reviewing 261 clinical records of individuals with suspected or present infection. Univariate, bivariate, and multivariate analyses were performed. 91.2% of the individuals were men, and 8.8% were women, with the mean age being 28.61 (SD = 9.35, Range = 17–74) years old. The prevailing sexual orientation identity was homosexual. 94.2% were single. The main reason for consultation was HIV. Differences in prevalence were found between crisis and non-crisis years (OR = 3.91; 95% CI = 1.73–9.19). In conclusion, their profile was that of a young, single man suspecting possible HIV infection. STI prevalence was significantly higher in the years of economic recession in comparison to the rest of the years.

## 1. Introduction

Sexually Transmitted Infections (STIs) are a major public health issue, both due to their morbidity rates and the complications and sequelae associated with them. Recent studies have noted the existence of certain groups that are particularly vulnerable to STIs, such as immigrants, adolescents, sex workers, men who have sex with men (MSM), and bisexuals [1,2]. MSM are a special interest group because of the increase in the prevalence of HIV and other STIs in recent decades [3].

The latest data published by the European Centre for Disease Prevention and Control (ECDC) for the years 2016 (syphilis) and 2017 (congenital syphilis, gonorrhoea, chlamydia trachomatis, and lymphogranuloma venereum) report an increase in these infections in different population groups. More specifically, in the case of syphilis, 66% of the new cases reported, in which the transmission category is provided, were in MSM [4]. Almost all of the reported cases of lymphogranuloma venereum were in MSM [5]. 10% of the reported cases of chlamydia infections were in MSM [6]. 47% of the cases of gonorrhoea infection were in MSM [7]. Finally, with respect to HIV, transmission in MSM was the most frequent, accounting for 54.3% of all reported cases [8].

The number of new HIV diagnoses in MSM continues to rise in the United States. In 2016, these diagnoses accounted for 82% of new diagnoses, with the age group at highest risk of new diagnosis being those between 13 and 24 years old, this group having experienced an increase of 24% in the number of new diagnoses since 2010 [9]. Syphilis cases also show a steady increase since 2008 in MSM, according to a study conducted in 20 U.S. cities [10].

Certain properties of an individual’s sexual behaviour, such as levels of promiscuity, early first sexual intercourse, number of sexual partners, and correctness of condom usage, determine the level of vulnerability in this group [11,12]. In addition, the following stand out: the use of alcohol and drugs, the use of the Internet and other new technologies to easily find and meet sexual partners, the optimism caused by the emergence of antiretroviral treatments, and the lack of effectiveness of prevention programmes [13,14,15,16,17,18,19].

The economic, cultural, and political situation has repercussions inth e area of public health and, more specifically, in the incidence of STIs. Spain endured an economic crisis between 2008 and 2014, in which some STIs, such as syphilis and gonorrhoea, which were considered virtually eradicated, resurfaced. In addition, the incidence of other infections, such as HIV, hepatitis, and Human Papillomavirus (HPV), also increased, when HPV control appeared to have been achieved [20].

Based on the above, the general objective of this study was to analyse the socio-demographic characteristics, and healthcare received, as well as the main risk behaviours in relation to STIs in the homosexual and bisexual population seen at the STI clinic in Granada during the years 2000–2015. During this period, some years have been characterised by a strong economic recession (2008–2014). Given the importance of this recession as a social determinant, the specific objective was thus to analyse the differences in the prevalence of these infections between this period and the rest of the years included in the study.

## 2. Materials and Methods

A cross-sectional observational analytic study was conducted by reviewing the medical records at the Sexually Transmitted Infection Clinic in Granada. A total of 261 cases of homosexual and bisexual individuals were analysed. These cases had been extracted from a larger sample of 1536 clinical records that were collected as part of a study which had been carried out by the research team since 2012.

For the selection of these clinical records, records of adult individuals without cognitive impairment who visited the clinic for suspicious reasons or the presence of an STI were considered. Individuals were identified as potential participants when a condition which suggested a possible future diagnosis was met, as stated in the record: symptoms, control, contact follow-up, and HIV.

The sample size was calculated to detect differences in a binary variable (in this case, presence, or absence of STI), seeking to detect differences of 20% in two years, with a statistical power of 80%, provided that the test was performed with an error of α = 5%. The number of clinical records needed per year was 97. In order to select the records, the first and last record numbers were taken from the archive of each year’s new records. Subsequently, an annual sample was extracted using systematic random sampling. The study period covers 15 years (2000 to 2015).

The variables collected were the following: socio-demographic (age, sex, nationality, occupation, employment status, level of education, marital status, sexual orientation identity); clinical care received (reason for visit, previous visit, number of subsequent visits, and number of new subsequent episodes); risk indicators (regular partner, period of time since last sexual contact without a condom, number of partners in the last month, number of partners in the last year, contact with a sex worker, regular partner having symptoms, drug use, frequency of drug use, previous STIs, and age of first sexual intercourse).

The following variables, registered in the clinical records as nominal variables, were transformed into ordinal variables for the ease of analysis: the period since last sexual contact without a condom; the number of partners in the last month; and number of partners in the last year. Similarly, the following variables were coded as binary for bivariate analysis: the level of education; marital status; and the reason for the visit.

STI diagnosis was included as the dependent variable and coded as binary (yes/no), following the pattern established by other studies in this line of research [21]. This variable was compared to the rest of the variables described above, which were considered to be independent variables for this analysis. Finally, in order to meet the specific objective, the records were grouped into two time periods: 2000–2007 and 2015, which correspond to the years of absence of the crisis or economic recession, and 2008–2014, which correspond to the years of recession, according to data from the Spanish Ministry of Economy and Business [22].

The data were gathered in a data collection sheet created specifically for this purpose and then transferred to a computerised database. In order to address the general objective of the study, the univariate analysis was carried out first. For quantitative variables, descriptive statistics were computed (mean, median, interquartile range, 95% confidence interval). For qualitative variables, absolute frequencies and percentages were calculated. Subsequently, bivariate analyses were carried out to compare the dependent variable with the independent variables. The Mann-Whitney *U*-test was used if the independent variable was quantitative. This non-parametric test was chosen due to the absence of normality of the analysed variables. This was verified by the Kolmogorov-Smirnov and Shapiro-Wilk tests, as well as by the ordinal nature of some study variables and the small sample size obtained in some comparison groups. For qualitative independent variables, the Chi-squared test (*χ*²) or the generalisation of Fisher’s exact test was used where applicable.

In order to address the specific objective of the study, the frequency and percentage of STI diagnoses in the crisis and non-crisis periods were first calculated. It was then determined whether there were significant differences in STI prevalence between the two periods using the Chi-squared test. Finally, a multiple logistic regression was performed to control for potential confounding factors, taking the presence or absence of STIs as the dependent variable and the crisis/non-crisis period as an independent variable. These factors were identified after comparing the samples from both study periods on the basis of the variables described above. The tests already described were used for the bivariate analyses. In order to measure the strength of the association, the odds ratio was calculated with its corresponding 95% CI. Once the regression model was generated, the fitting conditions were checked the: collinearity between variables was explored by calculating the Variance Inflation Factor (VIF); the linearity of the dependent variable was checked against the quantitative variables included in the model; calibration was determined by means of the Hosmer–Lemeshow test for goodness of fit; and discrimination was determined according to the value of the area under the ROC curve.

Univariate and bivariate analyses were conducted using the Statistical Package for the Social Sciences (SPSS) program, version 22, (IBM, New York, USA, for Windows). Multiple logistic regression was performed with the R Commander software, version 3.2.2, Free Software Foundation’s GNU General Public License, Project R-UCA in Spanish. The statistical significance threshold was set at *p* < 0.05.

Before this study was carried out, approval was obtained from the Biomedical Research Ethics Committee of the province of Granada and from the Management Directorate of the Granada-Metropolitano Health District, which is responsible for the STI clinic where the research was carried out. Patient data were handled with the utmost confidentiality and in compliance with the Spanish Organic Law 15/1999, of the 13th of December, on Personal Data Protection, and the Spanish Organic Law 3/2018, of the 5th of December, on Personal Data Protection and guarantee of digital rights.

## 3. Results

**Figure 1:**
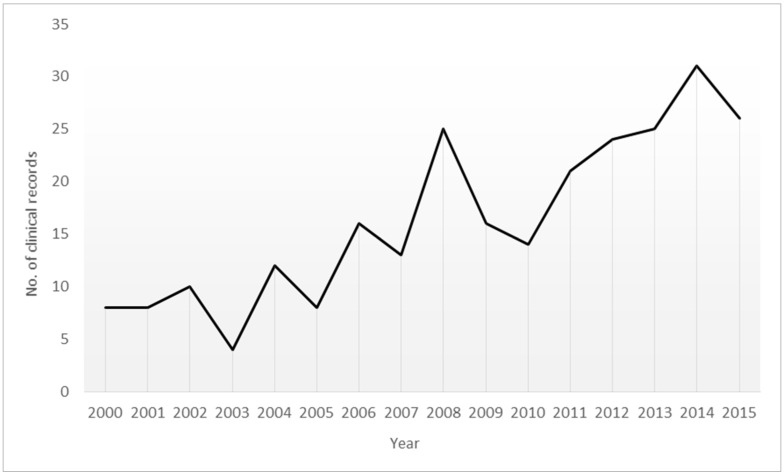
Progression of clinical records of the homosexual and bisexual population (2000–2015).

Figure 1 shows the progression of the number of records analysed in the sample that corresponded to homosexual and bisexual individuals.

Table 1 displays the characteristics of the sample in relation to the socio-demographic variables, healthcare received, and risk indicators.

STI diagnosis was recorded in 132 cases, with a negative diagnosis in 50 of them (37.9%) and a positive diagnosis in 82 of them (62.1%).

No statistically significant differences were found in this variable when compared to the rest of the variables (Table 2, Table 3 and Table 4).

**Figure 2:**
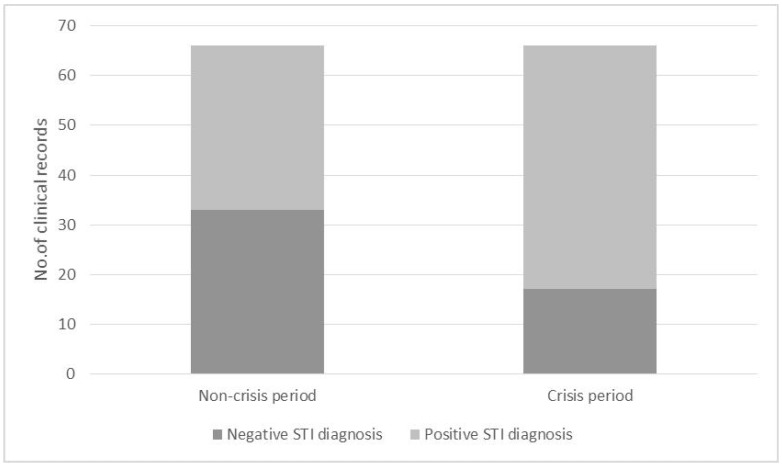
Sexually Transmitted Infections (STIs) diagnosis vs. Crisis/Non-crisis period.

When analysing the presence of STIs between the crisis and non-crisis periods, it was found that, during the non-crisis period, 50% of diagnoses were positive and 50% of diagnoses were negative (*n* = 33 in a sample of 66). In contrast, in the crisis period, the percentages were 74.24% (*n* = 49) for positive diagnoses, and 23.75% (*n* = 17) for negative diagnoses, also in a sample of 66 cases. There was an increase in the number of STIs diagnosed during the crisis period versus the non-crisis period, with this difference being statistically significant (*p* = 0.004) (Figure 2).

In order to analyse whether the statistical association observed could be conditioned by a possible confounding factor related to any of the variables described above, we compared potential confounds (socio-demographic variables and risk indicators) in the sample between both time periods.

The results showed that, in both periods, the populations were homogeneous in all of the variables compared, except for nationality (*p* = 0.002) and number of partners in the last month (*p* < 0.001), in which statistically significant differences were found.

After fitting this association with these two factors using logistic regression (Table 5), a statistically significant association was still observed (*p* = 0.001) with an odds ratio value (crisis/non-crisis period) of 3.91 (95% CI: 1.73–9.19).

## 4. Discussion

### 4.1. Main Findings

With regards to the number of homosexual and bisexual individuals who have visited the study clinic, the progressive increase in cases throughout the study period is noteworthy. This increase may be linked to the progressive reduction of stigma and social discrimination against these minority groups, which might lead to the increased self-determination of their sexual behaviour or sexual orientation and the public manifestation thereof. In spite of being in the midst of a process of change in the attitudes of the general population towards these communities, it should be pointed out that there is still a discriminatory attitude that perpetuates their vulnerability even more. Previous studies have highlighted the existing association between stigma and discrimination against these groups, including low self-esteem, depression, and substance use. All of this is conducive to risky sexual practice [13].

In the study period, in the analysed individuals who received their serological test results, a greater prevalence was observed in positive STI diagnoses in comparison to negative STI diagnoses. This finding is in consonance with a recent study in which 365 MSM were monitored, resulting in 253 individuals being diagnosed with one or more STIs during the first two years, with an incidence rate of 90.4 per 100 individuals per year. Other studies suggest that the issue of STIs in the MSM population has been increasing in recent decades, largely due to the risky behaviours adopted by this population [13], while mentioning improvements in biomedical HIV interventions as one of the factors influencing the adoption of risky behaviours [23].

In terms of healthcare indicators, the reason for visit relating to suspected HIV infection was the most common, followed by STI symptoms. It is noteworthy that three-quarters of the sample reported no previous STIs and that the majority of the individuals did not make a previous visit due to suspected STIs. This illustrates the role of these specialised clinics as referral centres for addressing this health issue in this population group [11].

Drug use is a risk factor reported by other investigations which indicate that risky practices are often related to drug use and to certain places of sexual contact, such as private parties, clubs, and saunas [24]. The results found in the present study are not significant in this sense, but a trend can certainly be observed in this respect.

Another risk indicator analysed was the period since the last sexual contact without a condom, with data pointing to inconsistent and infrequent condom use. With respect to the number of partners in the last month and in the last year, the data extrapolated from the clinical records yield a value of between 1–2 partners in the last month and 5–10 in the last year. Both inconsistent condom use and having a large number of sexual partners have been described by other studies as predictors of STI risk, mainly in the adolescent population [24,25].

The age of first sexual intercourse was around the age of 17 years. Other authors [12,26] point to the beginning of sexual relations at even earlier ages, around 15 years old. It is well known that an early onset in this type of relation promotes the occurrence of risky sexual behaviours, as well as an increased risk of contracting STIs [11].

Finally, regarding the specific objective, it should be noted that there was an increase in the prevalence of STIs during the crisis period in comparison to the non-crisis period. This finding is consistent with a previous study by the authors [27] which, unlike the present research, was conducted on the general population and covered a shorter period. In line with the contributions of other authors [28,29], the negative effect of financial crises on infectious conditions is particularly noteworthy. Greece, one of the European countries that has suffered most from the financial crisis, is a prime example of this, where several studies [30,31] have revealed an increase in prevalence of several infectious conditions, including HIV, pointing to budget cuts and the dismantling of a third of all EU prevention programmes between 2009–2010 as possible causes [31]. Interestingly, one of the studies published on the economic crisis and communicable diseases in Europe [32] highlights how STIs and vulnerable groups, such as immigrants, drug users, homeless people, and MSM would be affected.

### 4.2. Limitations

Among the limitations of the present study, first of all, is the fact that the results cannot be extrapolated to the general population of homosexuals and bisexuals since this study was carried out in a single clinic. Of the total number of records collected for the research project, of which this study forms a part, the sample of homosexual and bisexual individuals accounted for 17%. According to a survey carried out in several European countries, 14% of Spanish people between the ages of 14 and 29 would identify themselves as lesbian, gay, bisexual or transgender (LGBT) [33]. Taking into account that the age of the individuals in the records analysed was around 26 and 29 years old, it is fairly safe to conclude that the representation obtained is equivalent to that observed in the general population. However, it should be kept in mind that the distribution by sex differs from the aforementioned survey, with men being more represented than women. It should also be taken into consideration that our sample focuses mainly on men who identify as homosexual or bisexual, whereas the scientific literature consulted refers to MSM, who may view themselves as heterosexual, while still including homosexual and bisexual men.

In addition, being a specialised clinic, the subjects who visit it are attributed to risky behaviour for merely visiting it. In this sense, an underreporting of certain behaviours due to the effect of social desirability cannot be ruled out either, as the clinical records are completed by means of a personal interview.

The percentage of values missing in some of the variables should be taken into account. In some cases, this absence responds to the lack of completion of the clinical record, which could not be controlled in this study. In other cases; however, these were values that were not suitable for collection due to the profile of the individual studied. For this reason, it was preferred to carry out an analysis of the complete cases per variable, showing the sample size analysed in each variable.

Another limitation has to do with the type of design used. In spite of analysing a wide time series, since it is a cross-sectional study, the associations found can only be considered to be causal hypotheses.

### 4.3. Implications for Practice and Research

The results of this research would reinforce the idea of the need to develop education programmes for the prevention of STIs, especially in vulnerable groups, such as homosexual and bisexual populations, that inform and provide tools on what these infections imply and promote the adoption of attitudes, strategies, and personal behaviours that enable these populations to protect themselves from STIs. Emphasis should be placed on prioritising their initiation in these health education programmes from an early age before the first risky behaviours begin. In addition, health policy actions are needed to strengthen specialised STI care and work on prevention through the Internet [23].

In line with the above, one aspect that has not been dealt with in this research, but which will be the subject of future studies, has to do with the use of new technologies through the Internet to search for sexual partners. Increased use of these technologies, especially among younger people, has been demonstrated in other studies reviewed, which reported that young MSM currently meet their first sexual partners through the Internet [34]. Other studies conclude that this practise should be considered to be a risk factor for contracting STIs and HIV [19,35].

The study involved a low number of female individuals. It is, therefore, necessary to conduct future studies that include more women or that are exclusively developed on a female population.

In addition, further longitudinal studies should be carried out to establish more solid causal relationships than those observed in this research, as well as qualitative studies to determine the reasons associated with risky sexual practices from the perspective of homosexual and bisexual individuals.

## 5. Conclusions

As a conclusion, during the period 2000–2015, the profile of the individuals who visited the sexually transmitted infection clinic were mostly young homosexual men of Spanish nationality whose predominant marital status was single. The main reasons for the visit were the suspicion of HIV infection and STI symptoms, with a positive STI diagnosis prevailing when a serological test was performed. No statistically significant differences were found in STI diagnoses when other factors were compared, such as socio-demographic factors, factors relating to the healthcare received, and risk indicators. Differences in STI prevalence were found between crisis and non-crisis periods, with increased STI prevalence during the crisis period.

## Figures and Tables

**Table 1:** Sample characteristics.

**Socio-Demographic Data**				
	**Mean**	**95% CI**	**Me**	**IQR**
Age (*n* = 261)	28.61	24.47–29.75	26.00	10
	*n*		%	
Sex (*n* = 261)				
Male	238		91.2%	
Female	23		8.8%	
Nationality (*n* = 258)				
Spanish	230		89.1%	
Non-Spanish	28		10.9%	
Occupation (*n* = 250)				
Other occupations/Unpaid occupation	126		50.4%	
Student	124		49.6%	
Employment status (*n* = 244)				
Employed	91		37.3%	
Unemployed	25		10.2%	
Retired	4		1.6%	
Student	124		50.8%	
Level of education (*n* = 253)				
No education	1		0.4%	
Primary/Elementary/Basic education	13		5.1%	
Secondary education	46		18.2%	
Vocational training	33		13.0%	
Higher education	160		63.2%	
Marital status (*n* = 258)				
Single	243		94.2%	
Married/Common-law marriage	9		3.5%	
Separated/Divorced	6		2.3%	
Sexual orientation identity (*n* = 261)				
Bisexual	57		21.8%	
Homosexual	204		78.2%	
**Clinical care received**				
	*n*		%	
Reason for visit (*n* = 261)				
Symptoms	75		28.7%	
Control	14		5.4%	
Contact follow-up	2		0.8%	
HIV	170		65.1%	
Previous visit (*n* = 211)				
Yes	52		24.6%	
No	159		75.4%	
	**Mean**	**95% CI**	**Me**	**IQR**
No. of subsequent visits (*n* = 260)	1.19	1.06–1.33	1.00	0
No. of new subsequent episodes (*n* = 259)	0.69	0.54–0.84	0.000	1
**Risk indicators**				
	***n***		**%**	
Has regular partner (*n* = 244)				
Yes	123		50.4%	
No	121		49.6%	
Contact with sex worker (*n* = 126)				
Yes	8		6.3%	
No	118		93.7%	
Regular partner has symptoms (*n* = 76)				
Yes	39		51.3%	
No	37		48.7%	
Uses drugs (*n* = 165)				
Yes	50		30.3%	
No	115		69.7%	
Frequency of drug use (*n* = 47)				
Usually	14		29.8%	
Sporadically	31		66.0%	
Not currently	2		4.3%	
Previous Sexually Transmitted Infections (STIs) (*n* = 217)				
Yes	54		24.9%	
No	163		75.1%	
	**Mean**	**95% CI**	**Me**	**IQR**
Period since last sexual contact without a condom (*n* = 184)	2.62	2.49–2.75	3.00	1
No. of partners in the last month (*n* = 244)	1.59	1.48–1.71	1.00	1
No. of partners in the last year (*n* = 241)	3.13	2.95–3.32	3.00	2
Age of first sexual intercourse (*n* = 172)	17.76	17.29–18.22	17	3

*n* = sample size; 95% CI = 95% Confidence Interval; Me = Median; IQR = Interquartile Range; Period of time since last sexual contact without a condom: 1 = never, 2 = less than one month, 3 = one to six months, 4 = six to 12 months, 5 = more than 12 months; No. of partners in the last month: 1 = 0–1, 2 = 2, 3 = 3–5, 4 = more than 5; No. of partners in the last year: 1 = 0–1, 2 = 2, 3 = 3–5, 4 = 6–10, 5 = 11–20, 6 = more than 20.

**Table 2:** STI diagnosis vs. Socio-demographic characteristics.

Variables	Negative STI Diagnosis					Positive STI Diagnosis					*p*
	*n*	Mean	Me	95% CI	IQR	*n*	Mean	Me	95% CI	IQR	
*Age* (*n* = 132)	50	31.38	28	27.84–34.92	14	82	28.40	26	26.48–30.33	10	*ns*
	*n*		%			*n*		%			*p*
Sex (*n* = 132)											
Male	44		37.0%			75		63.0%			*ns*
Female	6		46.2%			7		53.8%			
Nationality (*n* = 132)											
Spanish	46		38.3%			74		61.7%			*ns*
Non-Spanish	4		33.3%			8		66.7%			
Occupation (*n* = 126)											
Other occupations/Unpaid occupation	24		33.8%			47		66.2%			*ns*
Student	22		40.0%			33		60.0%			
Employment status (*n* = 124)											
Employed	19		38.0%			31		62.0%			*ns*
Unemployed	4		25.0%			12		75.0%			
Retired	2		66.7%			1		33.3%			
Student	22		40.0%			33		60.0%			
Level of education (*n* = 126)											
Higher education	33		37.9%			54		62.1%			*ns*
Others	15		38.5%			24		61.5%			
Marital status (*n* = 131)											
Single	44		36.4%			77		63.6%			*ns*
Others	5		50.0%			5		50.0%			
Sexual orientation identity (*n* = 132)											
Bisexual	16		50.0%			16		50.0%			*ns*
Homosexual	34		34.0%			66		66.0%			

*n* = sample size; 95% CI = 95% Confidence Interval; Me = Median; IQR = Interquartile Range; *p* = *p*-Value; ns = not significant.

**Table 3:** STI diagnosis vs. Healthcare received.

Variables	Negative STI Diagnosis					Positive STI Diagnosis					***p***
	*n*		%			*n*		%			
Reason for visit (*n* = 32)											
Others	33		39.8%			50		60.2%			*ns*
HIV	17		34.7%			32		65.3%			
Previous visit (*n* = 110)											
Yes	10		24.4%			31		75.6%			*ns*
No	28		40.6%			41		59.4%			
	*n*	Mean	Me	95% CI	IQR	*n*	Mean	Me	95% CI	IQR	*p*
No. of subsequent visits (*n* = 131)	49	0.92	1.00	0.63–1.20	1	82	1.38	1.00	1.07–1.69	2	*ns*
No. of new subsequent episodes (*n* = 131)	49	0.57	0.00	0.32–0.83	1	82	1.09	0.00	0.77–1.40	2	*ns*

*n* = sample size; *p* = *p*-Value; Me = Median; 95% CI = 95% Confidence Interval; IQR = Interquartile Range; ns = not significant.

**Table 4:** STI diagnosis vs. Risk indicators.

Variables	Negative STI Diagnosis					Positive STI Diagnosis					*p*
	*n*		%			*n*		%			
Regular Partner (*n* = 122)											
Yes	26		43.3%			34		56.7%			*ns*
No	21		33.9%			41		66.1%			
Contact with sex worker (*n* = 66)											
Yes	2		40.0%			3		60.0%			*ns*
No	21		34.4%			40		65.6%			
Regular partner having symptoms (*n* = 36)											
Yes	9		56.25%			7		43.75%			*ns*
No	6		30.0%			14		70.0%			
Uses drugs (*n* = 74)											
Yes	7		33.3%			14		66.7%			*ns*
No	25		47.2%			28		52.8%			
Frequency of drug use (*n* = 18)											
Usually	2		33.3%			4		66.7%			*ns*
Sporadically	5		41.7%			7		58.3%			
Previous STIs (*n* = 109)											
Yes	10		33.3%			20		66.7%			*ns*
No	32		40.5%			47		59.5%			
	*n*	Mean	Me	95% CI	IQR	*n*	Mean	Me	95% CI	IQR	*p*
Period since last sexual contact without a condom (*n* = 88)	35	2.71	3.00	2.36–3.07	1	5347	2.34	2.00	2.15–2.53	1	*ns*
No. of partners in the last month (*n* = 122)	47	1.70	2.00	1.47–1.94	1	75	1.72	1.00	1.50–1.94	1	*ns*
No. of partners in the last year (*n* = 120)	45	3.40	4	2.99–3.81	1	75	3.05	3	2.72–3.38	2	*ns*
Age of first sexual intercourse (*n* = 78)	33	18.39	18.0	17.05–19.74	5	45	17.40	17.0	16.42–18.38	2	*ns*

*n* = sample size; *p* = *p*-Value; Me = Median; 95% CI = 95% Confidence Interval; IQR = Interquartile Range; ns = not significant; Period of time since last sexual contact without a condom: 1 = never, 2 = less than one month, 3 = one to six months, 4 = six to 12 months, 5 = more than 12 months; No. of partners in the last month: 1 = 0–1, 2 = 2, 3 = 3–5, 4 = more than 5; No. of partners in the last year: 1 = 0–1, 2 = 2, 3 = 3–5, 4 = 6–10, 5 = 11–20, 6 = more than 20.

**Table 5:** Logistic regression for STI diagnosis vs. Crisis.

Variables	Crude OR	Adjusted OR (95% CI)	*p*	VIF
*Crisis*			0.001	1.16
Yes	2.88 (1.40–6.10)	3.91 (1.73–9.19)		
No	Ref.	Ref.		
*Nationality*			0.680	1.11
Non-Spanish	1.24 (0.37–4.87)	1.35 (0.32–6.07)		
Spanish	Ref.	Ref.		
*No. of partners in the last month*	1.02 (0.68–1.55)	1.27 (0.80–2.09)	0.317	1.19

OR = Odds Ratio; 95% CI = 95% Confidence Interval; VIF = Variance Inflation Factor; Calibration using the Hosmer–Lemeshow goodness-of-fit test: χ^2^ = 1.5644, df = 8, *p* = 0.991; Discrimination according to the ROC curve: area under the ROC curve with a value of 0.67 (95% IC = 0.57–0.75).
